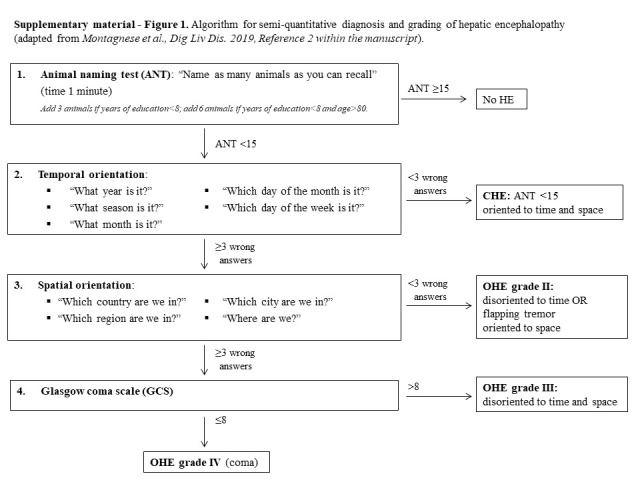# Overt Hepatic Encephalopathy: More Than Meets the Eye

**DOI:** 10.14309/ajg.0000000000002795

**Published:** 2024-04-08

**Authors:** Davide Erminelli, Chiara Mangini, Lisa Zarantonello, Paolo Angeli, Sara Montagnese

**Affiliations:** 1 Department of Medicine, University of Padova, Padova, Italy;; 2 Chronobiology Section, Faculty of Health and Medical Sciences, University of Surrey, Guildford, UK.

**Keywords:** cirrhosis, hepatic encephalopathy, neuropsychology, electroencephalography, ammonia

## Abstract

**INTRODUCTION:**

We aimed to assess the reliability of a qualitative approach to overt hepatic encephalopathy (OHE) diagnosis compared with the semiquantitative, and recommended one.

**METHODS:**

The above 2 methods were compared in 411 outpatients (71% males, 60 ± 10 years, model for end-stage liver disease 13.5 ± 5.0).

**RESULTS:**

Of the 73 patients with OHE on quantitative assessment, 19 (26%) were missed on qualitative assessment, with no difference in the likelihood of the physician missing grades II or III. Sixty-eight (20%) of the 270 patients with no OHE on quantitative assessment were wrongly qualified as having OHE.

**DISCUSSION:**

Qualitative clinical evaluation of OHE is not reliable, and recommendations should be followed.

## Graphical abstract

**Figure:**
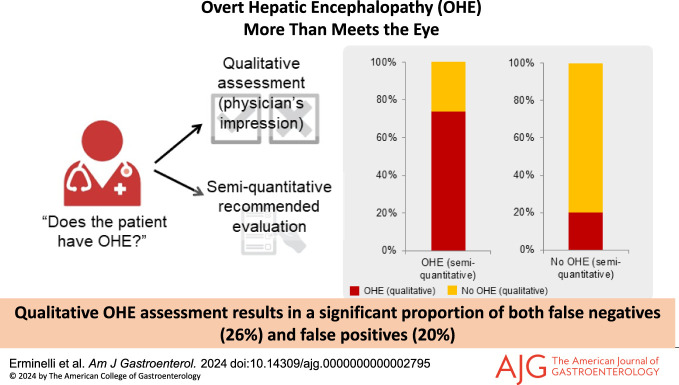

## INTRODUCTION

Although a semiquantitative assessment of overt hepatic encephalopathy (OHE) has been repeatedly and formally recommended (1,2), the impact of its replacement by unstructured interviews and/or physicians' impressions, which are still common practice, has never been formally assessed. The concept behind the recommendation is that verbal abilities tend to be preserved even in severe OHE (3,4), which can therefore be easily missed in conversation, unless questions ascertaining temporal and spatial orientation are actually asked.

The aim of this study was to assess the reliability and the impact, if any, of a qualitative approach to OHE diagnosis compared with the semiquantitative one recommended in the most recent set of Italian guidelines (2) (see Supplementary Figure 1, Supplementary Digital Content 1, http://links.lww.com/AJG/D241).

## METHODS

A total of 411 patients (71% males, 60 ± 10 years of age) evaluated in our dedicated HE clinic between April 2009 and June 2023 were included.

Before any formal assessment, patients were classified as having/not having OHE based on a qualitative impression of the physician—generally, a senior resident (i.e., a fourth- to fifth-year hepatology specialist trainee) or a consultant hepatologist—who clerked them into clinic, took their history, and went on to examine them. The doctor ticked *yes* or *no* in response to the question: *Does the patient have overt hepatic encephalopathy?* on the outpatient clinic form, before any formal neuropsychiatric assessment.

Patients were then formally evaluated by neuropsychological tests and electroencephalography (EEG), as fully described in Mangini et al (5), and qualified as unimpaired, when they were clinically normal and both the psychometric hepatic encephalopathy score (also summarized as the so-called mean PHES Z-score (6,7)) and the EEG were normal, having covert HE (CHE) when they were clinically normal but the Psychometric Hepatic Encephalopathy Score (PHES) and/or the EEG were abnormal, or having OHE based on a recommended semiquantitative modification of the West Haven criteria (2). This includes orientation to time (5 questions) and space (4 questions) plus the Glasgow Coma Scale (GCS) (8). OHE was graded as II (oriented to space, disoriented to time, or presence of asterixis), III (disoriented to time and space with a GCS ≥8), and IV (disoriented to time and space with a GCS <8; coma) (2). From 2016 onward, the animal naming test (ANT) was also administered (9).

The semiquantitative and qualitative OHE diagnoses were compared, and patients qualified as true positives (TPs) when the impression of the physician was consistent with the formal diagnosis of OHE; false negatives (FNs) when the physician did not recognize OHE; false positives (FPs) when patients were qualified as having OHE by the physician, but they did not meet the formal diagnostic criteria; and true negatives (TNs) when neither the physician nor the formal criteria confirmed an OHE diagnosis.

Permission for retrospective data analyses was obtained from the local Ethics Committee.

Results are expressed as mean ± SD or as count and percentage, as appropriate. Comparisons were performed by ANOVA (*post hoc* Tukey test) or by χ^2^, as appropriate. Analyses were performed with the package Statistica, version 13.1 (Dell, Round Rock, TX).

## RESULTS

One hundred twenty-two (30%) patients were qualified as having OHE on qualitative assessment.

**Figure 1.**
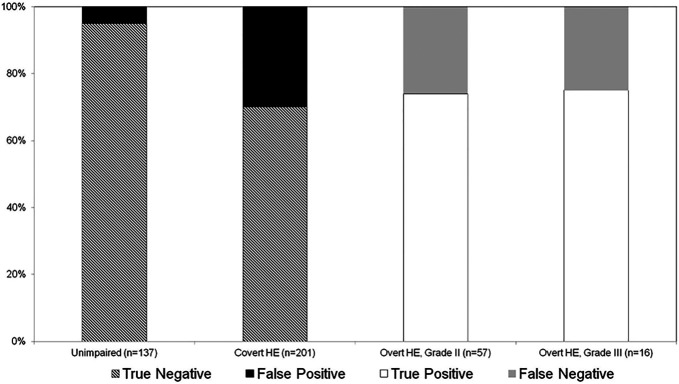
Percentage true-positive, false-negative, false-positive, and true-negative results, by degree of hepatic encephalopathy (HE).

On quantitative evaluation, 137 (33%) patients were qualified as unimpaired, 201 (49%) as having CHE, 57 (14%) as having grade II OHE, and 16 (4%) as having grade III OHE. Of the 73 patients with OHE, 19 (26%) were missed on qualitative assessment (FNs), with no difference in the likelihood of the physician missing grades II and III (Figure 1). Sixty-eight (20%) unimpaired/CHE patients were qualified as having OHE on qualitative assessment (FPs), of whom 61 (90%) had CHE and 7 (10%) were unimpaired (Figure 1).

**Figure 2.**
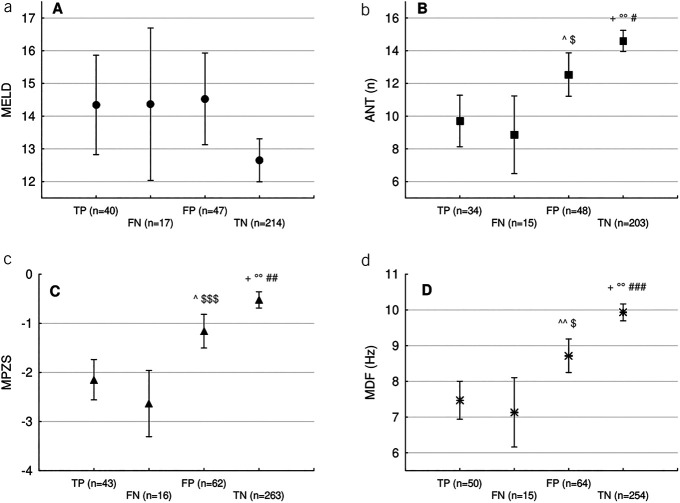
Mean ± 95% confidence interval of the variables: model for end-stage liver disease (MELD); (**a**) animal naming test (ANT, the higher the number of animals listed in 60 seconds, the better the performance); (**b**) mean Psychometric Hepatic Encephalopathy Score Z-score (MPZS, the higher the Z-score, the better the performance); (**c**) and the spectral EEG parameter mean dominant frequency (MDF, the higher the frequency, the better the EEG); (**d**) by classes of agreement between quantitative and qualitative assessment of hepatic encephalopathy. FN, false negatives; FP, false positives; TN, true negatives; TP, true positives. ^^^*P* < 0.05, ^^^^*P* < 0.01, significance of the difference between TP and FP; ^$^*P* < 0.05, ^$$$^*P* < 0.001, significance of the difference between FN and FP; ^+^*P* < 0.001, significance of the difference between TP and TN; ^°°^*P* < 0.001, significance of the difference between FN and TN; ^#^*P* < 0.05, ^##^*P* < 0.01, ^###^*P* < 0.001, significance of the difference between FP and TN on *post hoc* comparisons (Tukey test).

Demographic, hepatic failure, and neuropsychiatric features of the patients, by agreement between quantitative and qualitative HE assessment, are presented in Table 1 and Figure 2. The latter (Figure 2, panels b–d) highlights how FNs had virtually identical neuropsychiatric profiles as true positives. By contrast, FPs had slightly worse neuropsychiatric performances than TNs, suggesting that the physician was probably capable of detecting CHE. Finally, although overall model for end-stage liver disease scores were low (Table 1), they were even lower in TNs (Figure 2, panel a).

**Table 1.**
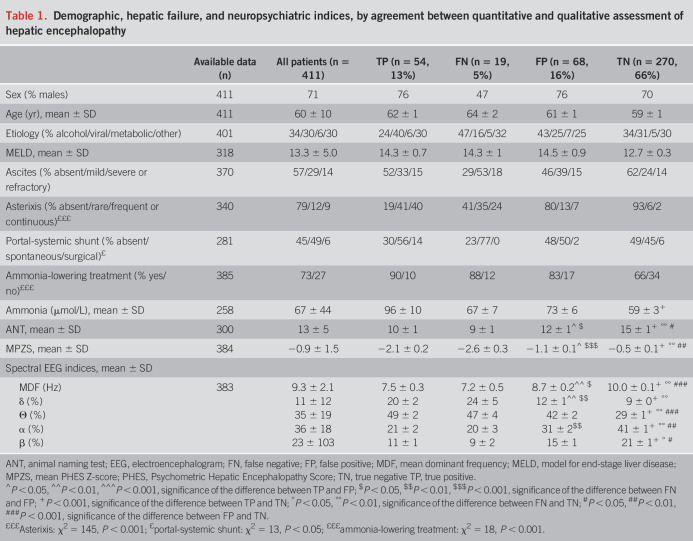
Demographic, hepatic failure, and neuropsychiatric indices, by agreement between quantitative and qualitative assessment of hepatic encephalopathy

## DISCUSSION

Qualitative clinical evaluation of OHE is not reliable, with approximately a quarter of patients with grades II and III OHE being missed. This confirms the appropriateness of the recommended evaluation tools and most likely also the theory behind them. The time taken to perform the semistructured interview, which is less than 2 minutes, seems a very reasonable investment by comparison with the missed diagnoses of grades II/III OHE patients, some of whom may deserve immediate management or hospitalization (10–12). By contrast, the large majority (90%) of FPs were CHE and not unimpaired, suggesting that the physician was actually capable of detecting a mild degree of neuropsychiatric impairment, which did not result in temporal disorientation or asterixis (2). Although the data on FPs may be confounded by the fact that the study was conducted in a tertiary referral center for hepatology with a research interest in HE, the data on FNs are even more worrying for the same reason. Neurologists and geriatricians need and use semiquantitative and quantitative tools to assess their patients' performance (13,14), and one cannot see why hepatogastroenterologists should not (15). In conclusion, although our data need to be replicated in multicenter studies, they support existing guidelines to routinely perform semiquantitative OHE assessment, which is clinically actionable and might reduce both underdiagnosis and overdiagnosis and treatment of OHE.

## CONFLICTS OF INTEREST

**Guarantor of the article:** Sara Montagnese, MD, PhD.

**Specific author contributions:** S.M.: study concept and design. D.E., C.M., L.Z., and S.M.: acquisition of data. D.E., C.M., L.Z., and S.M.: analysis and interpretation of data. D.E., C.M., and S.M.: drafting of the manuscript. P.A.: critical revision of the manuscript for important intellectual content. D.E., C.M., L.Z., and S.M.: statistical analysis. All authors have approved the final draft submitted.

**Financial support:** SM received a grant from Decompensated cirrhosis: identification of new combinatorial therapies based on system approaches (DECISION) Horizon 2020 (Call: H2020-SC1-BHC-2018-2020).

**Potential competing interests:** None to report.

**Data sharing statement:** The data that support the findings of this study are available on reasonable request from the corresponding author.

## Supplementary Material

**Figure:**